# Vortioxetine enhances the anti-pancreatic cancer efficacy of gemcitabine through reducing MAOB expression

**DOI:** 10.3389/fphar.2026.1856439

**Published:** 2026-09-08

**Authors:** Mengjie Gu, Yingxue Si, Kaizhi Luo, Chong Zhang, Tingting Xiong, Zhizhen Huang, Yijia Han, Xiaoli Zheng, Zejun Wang

**Affiliations:** 1 Hangzhou Linping District Integrated Traditional Chinese and Western Medicine Hospital, Teaching Hospital of Medical School,Hangzhou City University, Hangzhou, Zhejiang, China; 2 Department of Pharmacy, School of Medicine, Hangzhou City University, Hangzhou, Zhejiang, China

**Keywords:** epithelial-mesenchymal transition, gemcitabine, MAOB, pancreatic cancer, vortioxetine

## Abstract

**Introduction:**

Pancreatic cancer is a highly aggressive malignancy with a poor prognosis, largely owing to chemoresistance and frequent recurrence following gemcitabine-based therapy. This study investigated whether vortioxetine, a multimodal serotonin (5-HT) modulator, could enhance the antitumor efficacy of gemcitabine and explored the underlying molecular mechanisms.

**Methods:**

The effects of vortioxetine alone and in combination with gemcitabine were evaluated in pancreatic cancer cells and xenograft models. Cell proliferation, apoptosis, migration, invasion, and epithelial-mesenchymal transition (EMT) were assessed using functional assays and relevant molecular markers. RNA sequencing was performed to identify potential molecular targets of vortioxetine, followed by molecular docking, thermal stability assays, and gene knockdown experiments for mechanistic validation.

**Results:**

Vortioxetine significantly enhanced the inhibitory effects of gemcitabine on pancreatic cancer cell proliferation *in vitro* and tumor growth in xenograft models. The combination treatment markedly promoted apoptosis, as indicated by increased levels of cleaved poly(ADP-ribose) polymerase (PARP) and cleaved caspase-3. It also suppressed cancer cell migration and invasion and reversed EMT-related changes, as demonstrated by the upregulation of E-cadherin and downregulation of N-cadherin and Snail. RNA sequencing identified monoamine oxidase B (MAOB; EC 1.4.3.4) as a potential molecular target associated with vortioxetine treatment, which was further supported by molecular docking and thermal stability assays. Moreover, MAOB knockdown phenocopied the effects of vortioxetine by enhancing gemcitabine sensitivity and suppressing EMT-associated phenotypes.

**Discussion:**

Vortioxetine potentiates the antitumor efficacy of gemcitabine against pancreatic cancer, with its effects associated with reduced MAOB expression, enhanced apoptosis, and suppression of EMT. These findings support further investigation of vortioxetine combined with gemcitabine as a potential therapeutic strategy for overcoming chemoresistance in pancreatic cancer.

## 1 Introduction

Pancreatic cancer is a highly aggressive malignancy with poor prognosis, ranking fourth in global cancer-related mortality, and a dismal 5-year survival rate of only 12% (Siegel et al., 2023). The main pathological types include pancreatic ductal adenocarcinoma (PDAC), accounting for over 90% of cases with the worst prognosis, and pancreatic neuroendocrine tumors (PNETs), which exhibit more favorable outcomes. PDAC progression is characterized by genetic mutations such as KRAS (Duan et al., 2024), TP53 (Xie et al., 2014), CDKN2A (Goodwin et al., 2023), and SMAD4 ((Bl et al., 2009), (Wan et al., 2021)). Due to the insidious onset, most patients are diagnosed at advanced stages, with median survival of only 4–6 months (Ghaneh et al., 2023).

Chemotherapy remains the main treatment for advanced cases, with gemcitabine widely used as a first-line agent. Gemcitabine, a nucleoside analogue, inhibits DNA synthesis through incorporation into replicating DNA ((De Santis et al., 2012), (Kelley et al., 2023)). Gemcitabine is the backbone of several two-drug regimens, including combinations with nab-paclitaxel (GN), cisplatin (GP), capecitabine (GX), or S-1 (GS). However, the overall response rate of gemcitabine remains low, below 20%, with common problems of drug resistance and high recurrence rates. Multiple factors, including transport, activation, and metabolism-related enzymes, influence gemcitabine sensitivity. Combining gemcitabine with sensitizers, like 5-fluorouracil in FOLFIRINOX ((Labori et al., 2024), (Vivaldi et al., 2019)), improves outcomes and is standard practice (Nichetti et al., 2024). Nevertheless, the efficacy remains unsatisfactory, and overcoming resistance remains a key challenge. Identifying novel sensitizers targeting diverse resistance mechanisms could offer new therapeutic strategies to improve prognosis in pancreatic cancer.

5-HT (serotonin) is a monoamine neurotransmitter that plays a key role in regulating mood, behavior, metabolism, and cell proliferation. It influences various physiological processes, including pancreatic β-cell function (Paulmann et al., 2009), energy homeostasis (Crane et al., 2015; Yadav et al., 2010), and tumorigenesis (Li et al., 2023; Lv et al., 2020). Abnormal 5-HT signaling is implicated in cancer progression by promoting tumor growth (Alpini et al., 2008; Coufal et al., 2010; Nocito et al., 2008) migration, and metabolic reprogramming (Jiang et al., 2017; Saponara et al., 2018). Vortioxetine is a multimodal 5-HT modulator that inhibits serotonin reuptake and modulates multiple 5-HT receptor subtypes (Baldwin et al., 2016; Christensen et al., 2023). It has shown antitumor effects in gastric cancer. (Li et al., 2023; Lv et al., 2020). However, its role in pancreatic cancer remains largely unexplored. MAOB, a mitochondrial enzyme involved in neurotransmitter metabolism, is abnormally expressed in various tumors and may influence tumor behavior. The objective of this research was to explore the vortioxetine’s anti-pancreatic cancer activity and explores whether it is associated with reduced MAOB expression, aiming to provide new insights and therapeutic targets for pancreatic cancer treatment.

## 2 Materials and methods

### 2.1 Materials

Vortioxetine (ALD-V125545) was purchased from Aladdin (Shanghai, China). Gemcitabine (T0251) was obtained from Targetmol. 4% paraformaldehyde universal tissue fixative (BL539 A) was purchased from Biosharp (Beijing, China). Pre-stained protein marker (26,617) was obtained from Thermo Fisher Scientific (USA). Matrigel basement membrane matrix (356,234) was purchased from Corning (New York, USA). Trichloroacetic acid (81540C) was obtained from Adamas-beta (Shanghai, China). SRB powder (230,162) was purchased from Sigma-Aldrich (St. Louis, MO, USA). RIPA (strong) lysis buffer (P0013 B) was obtained from Beyotime (Shanghai, China). PMSF (A610425), Leupeptin (A600580), and Pepstatin (A610583) were purchased from Sangon Biotech (Shanghai, China). Antibodies against β-actin antibody (66009-1-Ig), E-cadherin antibody (20874-1-AP), N-cadherin antibody (22018-1-AP), and Cyclin B1 antibody (55004-1-AP) were purchased from Proteintech (Wuhan, China). Snail antibody (C15D3), Cleaved-PARP antibody (D64E10), and Cleaved-Caspase-3 antibody (9664S, 5A1E) were purchased from Cell Signaling Technology (Danvers, MA, USA). Primary antibody dilution buffer (RM02954) was obtained from ABclonal (Wuhan, China). SuperKine™ enhanced antibody dilution buffer (ATVF25051) and ECL kit (BMU102-CN) were purchased from Abbkine (Wuhan, China). Goat anti-Mouse IgG (H + L) HRP (111-035-003) and Goat anti-Rabbit IgG (H + L) HRP (115-035-003) were purchased from Jackson ImmunoResearch Laboratories (West Grove, PA, USA).

### 2.2 Cell lines and cell culture

The human pancreatic cancer cell lines Capan-1 (CL-0708) and BxPC-3 (CL-0042) were obtained from Procell Life Science & Technology Co., Ltd (Wuhan, China). The human pancreatic cancer cell line AsPC-1 (SCSP-5080) was purchased from the Cell Bank of the Chinese Academy of Sciences (Shanghai, China). The cell lines Capan-1, BxPC-3, and AsPC-1 were cultured in RPMI-1640 complete medium supplemented with 10% fetal bovine serum and 100 U/mL penicillin plus 0.1 mg/mL streptomycin (penicillin-streptomycin) in a humidified incubator at 37 °C with 5% CO_2_. Cells were passaged at a ratio of 1:2-1:4 when reaching 80%–90% confluence, and logarithmic-phase cells were used for experiments as needed. All cell lines were confirmed to be free of *mycoplasma* contamination.

### 2.3 Sulforhodamine B (SRB) assay

Capan-1, BxPC-3, and AsPC-1 pancreatic cancer cells were digested into single-cell suspensions and seeded in 96-well plates. After adherence, cells were treated with increasing concentrations of vortioxetine for 24, 48, or 72 h. Cell proliferation was assessed by SRB assay as previously described (Hu et al., 2020).

### 2.4 Wound healing assay

Capan-1, BxPC-3, and AsPC-1 pancreatic cancer cells were seeded in 24-well plates (8 × 10^4^, 1 × 10^5^, and 4 × 10^5^ cells/mL, respectively). After 24 h, cells were treated with vortioxetine alone or combined with gemcitabine. Mitomycin C (500 ng/μL) was added before scratch. A vertical wound was made with a 10 μL pipette tip, washed with PBS, and fresh medium with drug was added. Images were taken at 0, 24, 48, and 72 h, and wound closure was analysed.

### 2.5 Colony formation, migration and invasion assays

For colony formation assays, cells were seeded in 6-well plates (1-2 × 10^3^ cells/mL). After 24 h, cells were treated with control (DMSO), vortioxetine, gemcitabine, or their combination at specified concentrations. Medium with drugs was refreshed every 3 days. After 14 days, colonies were fixed, stained with 1% crystal violet, washed, dried, photographed, and counted.

Migration and invasion assays were performed using 24-well transwell chambers (8 μm pore size). Capan-1, BxPC-3, and AsPC-1 cells were seeded into the upper chamber in serum-free medium containing the indicated drugs. The lower chamber contained 0.6 mL medium with 20% FBS. Migration groups included vortioxetine gradients or combination treatments. For invasion assays, the upper chamber was pre-coated with Matrigel diluted in medium (1:10). After 24 h, migrated or invaded cells were stained with 0.5% crystal violet and counted under a light microscope.

### 2.6 Apoptosis analysis

For apoptosis analysis by flow cytometry, cells were seeded in 6-well plates and treated with vortioxetine, gemcitabine, their combination, or DMSO control for 48 h. Cells and supernatants were collected, washed twice with cold PBS, and resuspended in 1× Binding Buffer. Cells were stained with Annexin V-FITC and/or PI in the dark for 15 min at room temperature. After adding 400 μL Binding Buffer, samples were filtered through a 40 μm strainer and analysed by flow cytometry.

### 2.7 RNA sequencing analysis

BxPC-3 cells were treated with vortioxetine or vehicle control for 24 h (n = 3 independent biological replicates per group). Total RNA was extracted using TRIzol and quality-checked. Poly-A mRNA was enriched, fragmented, and reverse-transcribed into cDNA. Sequencing libraries (300 ± 50 bp) were prepared and sequenced on the Illumina NovaSeq 6,000 platform with an average depth of ∼30 million paired-end reads per sample. Raw reads were filtered using Fastp (v0.20.0), and clean reads were aligned to the human reference genome (hg38) using HISAT2 (v2.2.1). Read counts were quantified using FeatureCounts, and differential expression analysis was performed using DESeq2 (v1.32.0). Differentially expressed genes (DEGs) were screened using the criteria of FDR-adjusted P-value (padj) < 0.05 and |log2 Fold Change| > 2.

### 2.8 Real-time quantitative PCR (RT-qPCR)

Total RNA was isolated using TRIzol™ Reagent following standard procedures. RNA purity and concentration were checked by spectrophotometry. Reverse transcription was performed using HiScript II Q RT SuperMix, and qPCR was carried out with SYBR qPCR Master Mix according to the manufacturer’s protocols. β-Actin served as the internal control, and relative gene expression levels were determined using the 2^−ΔΔCT^ method. Specific primer sequences used in the study are provided in Table 1.

**TABLE 1:** The primers for qPCR amplification.

Application	Gene	Primer (5′-3′)
RT-qPCR	*β*-actin-F	CAT​GTA​CGT​TGC​TAT​CCA​GGC
​	*β*-actin-R	CTC​CTT​AAT​GTC​ACG​CAC​GAT
​	MAOB-F	CTG​GCA​GTC​AGA​ACC​AGA​GTC
​	MAOB-R	GAG​GGC​AAA​TGT​CTC​TCC​AA
​	HTR1B-F	GGG​TTC​CTC​AAG​CCA​ACT​TAT​C
​	HTR1B-R	GCC​AAT​AGC​ATA​ACC​AGC​AGT
​	SCN5A-F	CAC​GCG​TTC​ACT​TTC​CTT​C
​	SCN5A-R	CAT​CAG​CCA​GCT​TCT​TCA​CA
​	TERT-F	TCA​CGG​AGA​CCA​CGT​TTC​AAA
​	TERT-R	TTC​AAG​TGC​TGT​CTG​ATT​CCA​AT
​	TACR1-F	TGC​TGC​CTC​AAT​GAC​AGG​TGA
​	TACR1-R	CAT​TTA​GGA​TGG​CCG​CTT​GGC

### 2.9 Transfection

siRNAs (20 μM) were prepared by dissolving lyophilized powder in DEPC water and stored at −80 °C in 10 μL aliquots. Cells were seeded in 6-well plates and grown to 30%–40% confluence. For transfection, 4 μL siRNA (Table 2) was diluted in 200 μL jetPRIME buffer, mixed gently, incubated 5 min at room temperature, then 4 μL jetPRIME reagent was added and incubated for 15 min. The mixture was added to cells, which were cultured for 24–48 h before further assays.

**TABLE 2:** siRNAs sequences used in this study.

Name	Sequence (5′-3′)
siNC	UUC​UUC​GAA​CGU​GUC​ACG​UTT
siMAOB#1	GGA​CCA​ACC​CAG​AAU​CGU​ATT
siMAOB#2	CAC​GUU​GGA​UGA​UAC​CAA​ACC

### 2.10 Protein expression levels by western blotting

Cells were seeded in 6-well plates and treated with vortioxetine, gemcitabine, or their combination for 48 h. After collecting both supernatant and adherent cells, cells were washed with PBS and lysed using 10 μL RIPA lysis buffer per 1 × 10^5^ cells. After 30 min on ice with periodic vortexing, lysates were centrifuged at 12,000 rpm, 4 °C, for 30 min. Total protein was quantified using a BCA assay with BSA standards. Equal amounts of protein were mixed with loading buffer, denatured at 100 °C, separated by SDS-PAGE, and transferred onto PVDF membranes. Membranes were blocked with 5% milk, incubated overnight with primary antibodies at 4 °C, followed by HRP-labeled secondary antibodies, and visualized using ECL detection.

### 2.11 Molecular docking

Molecular docking and molecular dynamics (MD) simulations were performed to investigate the interaction between vortioxetine and the MAOB protein (PDB ID: 1S3E). The MAOB-vortioxetine complex was subjected to a 50 ns MD simulation. During the simulation, the root mean square deviation (RMSD) of the protein backbone and the ligand vortioxetine were calculated to evaluate the stability of the complex.

### 2.12 Cellular thermal shift assay (CETSA)

BxPC-3 and AsPC-1 cells (∼7 × 10^6^) were seeded in 100 mm dishes. After attachment, cells were treated with DMSO (control) or vortioxetine at 5× IC50 (45.15 μM for BxPC-3, 51.6 μM for AsPC-1) for 1–2 h at 37 °C. Cells were harvested, washed, resuspended in PBS with protease inhibitors, aliquoted (∼2 × 10^6^ cells/100 μL), and heated at gradient temperatures (40 °C–64 °C) for 3 min each. Samples were frozen in liquid nitrogen, thawed, centrifuged at 12,000 rpm (4 °C, 20 min), and supernatants collected for western blot analysis.

### 2.13 Animal experiments

BxPC-3 cells (5 × 10^7^) were suspended in 0.1 mL PBS and subcutaneously injected into right flank of nude mice. When tumors reached ∼80 mm^3^, 20 mice were randomized into four groups (n = 5 individual mice per group): control (vehicle, 0.1 mL/10 g, i. p.), vortioxetine (15 mg/kg, oral, daily), gemcitabine (30 mg/kg, i. p., every 4 days), and combination. Body weight and tumor volume were recorded every 2 days. Mice were euthanized on day 24. Tumor volume = (length × width × height)/2, RTV = Vt/V0. Since each mouse was inoculated with a single tumor, each animal represents a single, statistically independent biological replicate (n = 5 per group).

### 2.14 Statistical analyses

All quantitative data are presented as mean ± standard deviation (SD). *In vitro* experiments were performed with at least three independent biological replicates unless otherwise stated. For animal experiments, five mice were included in each group. Statistical analyses were performed using GraphPad Prism. Data normality was assessed using the Shapiro–Wilk test, and homogeneity of variance was evaluated using the Brown–Forsythe test. For comparisons among multiple groups with one independent variable, one-way analysis of variance (ANOVA) followed by Tukey’s multiple-comparison test was used. For experiments involving two independent variables, such as treatment and time or treatment and drug concentration, two-way ANOVA followed by Šídák’s multiple-comparison test was applied. When the assumptions of equal variance were not met, Welch’s ANOVA followed by Dunnett’s T3 multiple-comparison test was used. P values were adjusted for multiple comparisons where appropriate. Statistical significance was defined as P < 0.05. Significance levels were indicated as follows: *P < 0.05, **P < 0.01, ***P < 0.001, and ****P < 0.0001.

## 3 Results

### 3.1 Vortioxetine moderately suppresses the progression of pancreatic cancer cells

**FIGURE 1:**
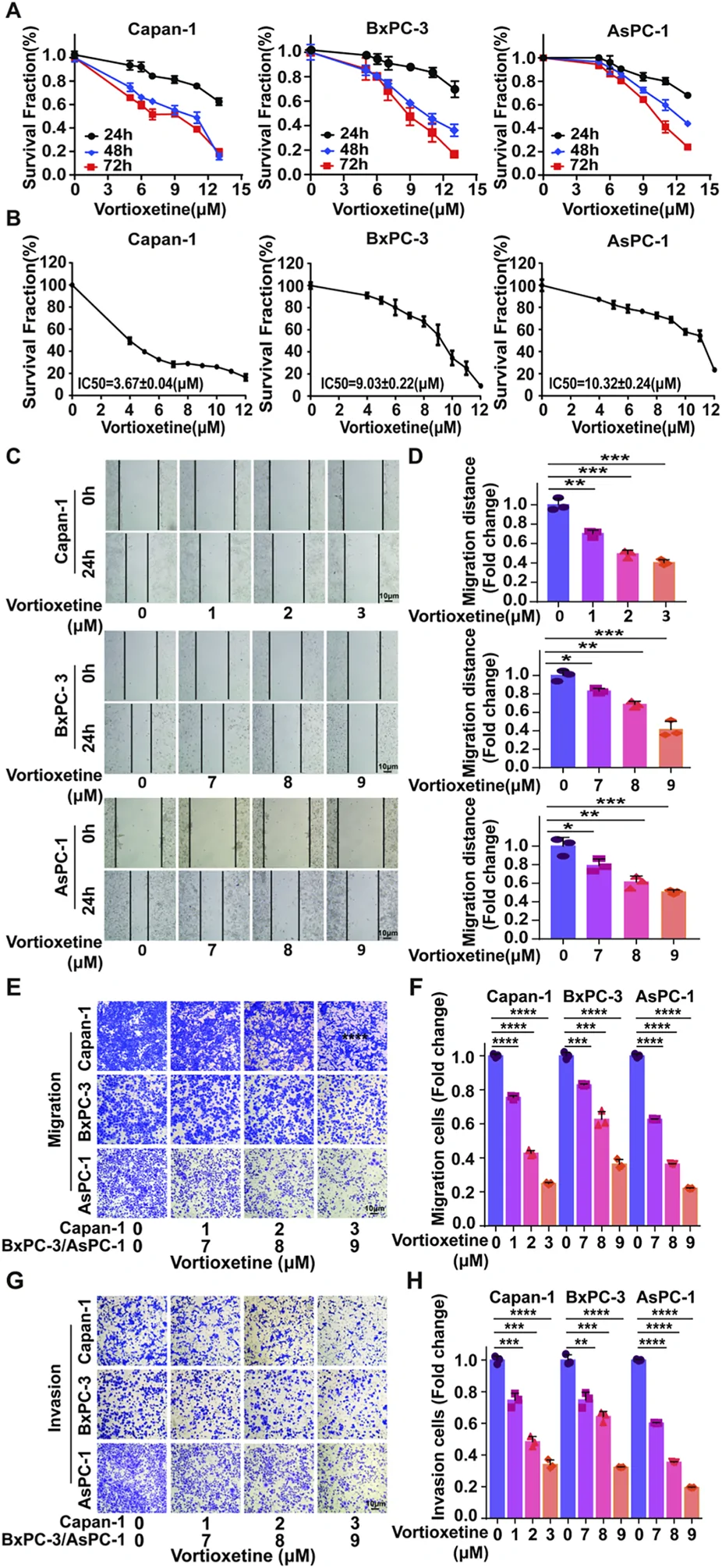
The effect of vortioxetine in human pancreatic cancer cells. **(A)** The proliferation inhibition curve of Capan-1 cells, BxPC-3 cells, and AsPC-1 cells. **(B)** IC50 values of vortioxetine in human pancreatic cancer cells. Vortioxetine inhibits the wound-healing ability of pancreatic cancer cells. **(C)** Compared with the control group, each experimental group significantly inhibited the migration ability of pancreatic cancer cells. **(D)** Statistical analyses corresponding to **(C)** respectively. **(E–H)** Vortioxetine inhibited the migration and invasion of pancreatic cancer cells. F and H panels show the statistical analyses corresponding to E and G, respectively. In A, D, F, and **(G)**. Data are presented as mean ± SD from three independent biological replicates (n = 3). Statistical significance was determined by one-way ANOVA followed by Tukey’s multiple-comparison test. *P < 0.05, **P < 0.01, ***P < 0.001, ****P < 0.0001.

To investigate the effect of vortioxetine on the proliferation of pancreatic cancer cells, we employed the SRB assay to assess its cytotoxicity against human pancreatic cancer cell lines Capan-1, BxPC-3, and AsPC-1. Cells were treated with increasing concentrations of vortioxetine (5, 6, 7, 9, 11, and 13 μM) and cell viability was measured at 0, 24, 48, and 72 h by recording absorbance at 540 nm. As shown in Figure 1A, cell viability decreased in a dose- and time-dependent manner in all three cell lines. Based on these findings, we further determined the IC50 values of vortioxetine at 72 h, the most significant time point. The IC50 values were 3.67 ± 0.42 μM for Capan-1, 9.03 ± 0.21 μM for BxPC-3, and 10.32 ± 0.91 μM for AsPC-1 (Figure 1B), indicating variable sensitivity, with Capan-1 being the most sensitive. To assess the impact of vortioxetine on migration and invasion, cells were pretreated with mitomycin C (500 ng/μL) to inhibit proliferation before scratch and transwell assays. Scratch assays showed that vortioxetine significantly suppressed wound healing in a dose-dependent manner (*P < 0.05, **P < 0.01, ***P < 0.001, Figure 1C). Consistent results were observed in transwell migration assays (Figures 1E,F), where higher concentrations of vortioxetine led to more pronounced inhibition. Furthermore, transwell invasion assays confirmed that vortioxetine also suppressed the invasive capacity of all three cell lines, with the highest doses showing the strongest inhibitory effects (**P < 0.01, ***P < 0.001, ****P < 0.0001, Figures 1G,H). Due to the IC50 values of vortioxetine alone ranging from 3 to 11 μM in different pancreatic cancer cell lines, it exhibited moderate cytotoxicity and significantly inhibited migration and invasion only at higher concentrations, indicating potential for enhanced antitumor activity.

### 3.2 Vortioxetine enhances gemcitabine sensitivity in pancreatic cancer cells

**FIGURE 2:**
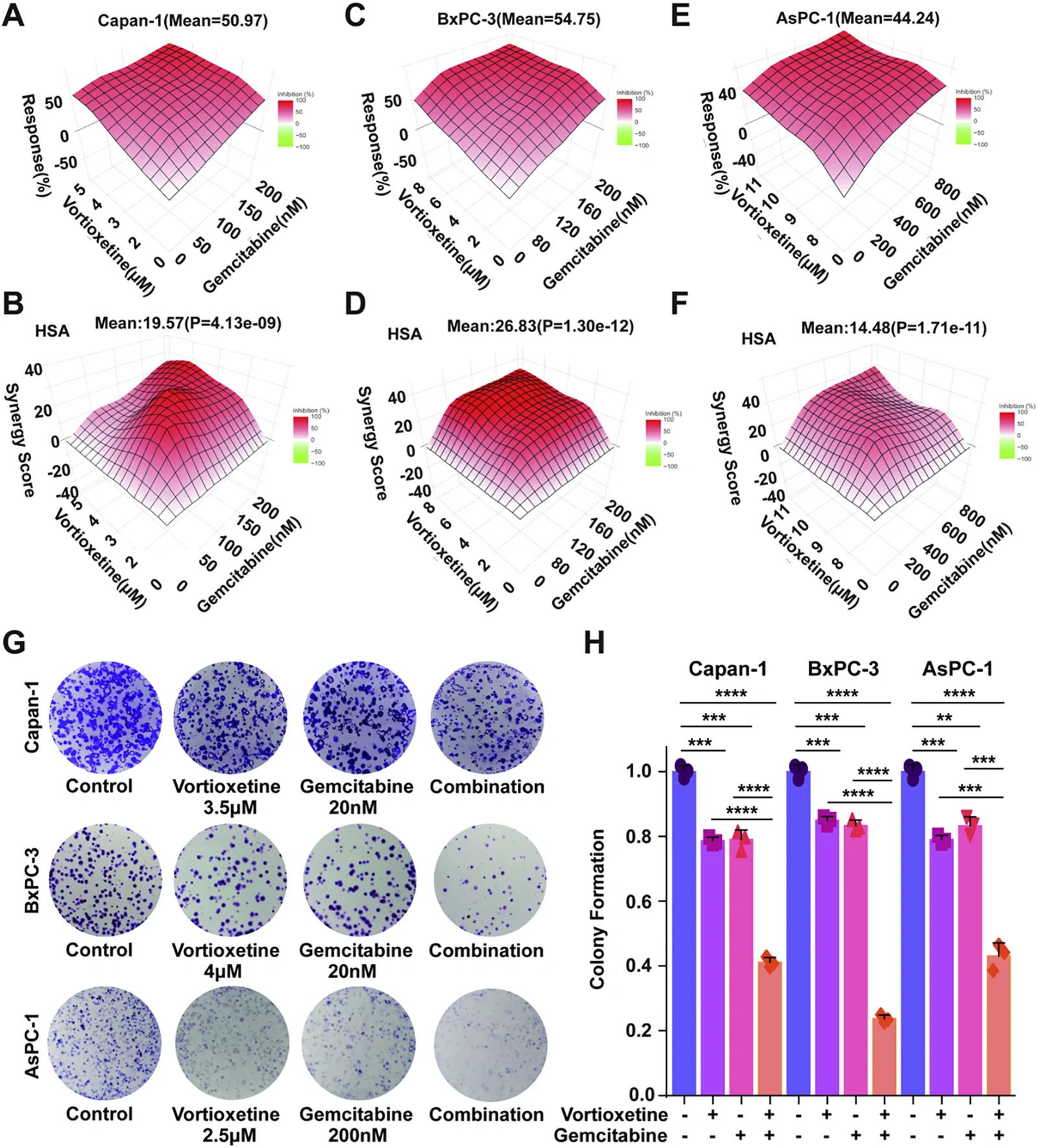
The inhibitory effect of vortioxetine combined with gemcitabine on the proliferation of pancreatic cancer cells. **(A,C,E)** The cell survival rates at various points corresponding to different gradient concentrations of vortioxetine alone or in combination with gemcitabine in Capan-1, BxPC-3 and AsPC-1 cells, where Response (%) indicates the overall impact of the drug combination on cell proliferation. **(B,D,F)** HSA (Highest Single Agent) values and Mean values corresponding to the scoring results of Capan-1 cells, BxPC-3 and AsPC-1 cells. **(G)** After treatment with vortioxetine alone or in combination with gemcitabine for 14 days, crystal violet staining photomicrographs of Capan-1, BxPC-3 and AsPC-1 cells were obtained. **(H)** Statistical analysis results of clone counting for Capan-1, BxPC-3 and AsPC-1 cells. In A, B, C, D, E, F, and **(H)**. Data are presented as mean ± SD from three independent biological replicates (n = 3). Statistical significance was determined by one-way ANOVA followed by Tukey’s multiple-comparison test. **P < 0.01, ***P < 0.001,****P < 0.0001.

Gemcitabine-based combination therapies, such as GN, GP, GX, and GS, are first-line regimens for pancreatic cancer. Considering gemcitabine resistance is a major challenge in treatment, we investigated whether combining vortioxetine could enhance gemcitabine sensitivity. SRB assays showed that the combination of vortioxetine and gemcitabine significantly reduced cell viability in BxPC-3, Capan-1, and AsPC-1 cells compared to either drug alone (Tables 3–5), suggesting synergistic effects. Using the SynergyFinder platform, we calculated HSA (Highest Single Agent) and Mean synergy scores. In BxPC-3 cells, HSA was 10.23 and Mean 16.04 (P = 4.97e-4), with Mean > HSA indicating synergy (Figures 2C,D). Similar synergy was observed in Capan-1 (HSA = 6.29, Mean = 8.92, P = 3.58e-4, Figures 2A,B) and AsPC-1 cells (HSA = 11.34, Mean = 17, P = 5.02e-3, Figures 2E,F).

**TABLE 3:** Survival fraction of Vortioxetine and/or gemcitabine in Capan-1.

Vortioxetine		Gemcitabine		Combination
Conc. (μM)	SF (%)	Conc. (nM)	SF (%)	SF (%)
2	70.29% ± 0.12%	50	89.79 ± 0.13	64.58 ± 0.18
3	54.54% ± 0.20%	100	81.52 ± 0.21	47.13 ± 0.12
4	46.98% ± 0.14%	150	62.41 ± 0.17	31.09 ± 0.21
5	36.71% ± 0.12%	200	40.33 ± 0.12	21.12 ± 0.15

**TABLE 4:** Survival fraction of Vortioxetine and/or gemcitabine in BxPC-3.

Vortioxetine		Gemcitabine		Combination
Conc. (μM)	SF (%)	Conc. (nM) (nM) (nM)	SF (%)	SF (%)
3	92.14 ± 0.23	20	89.99 ± 0.11	62.24 ± 0.17
4	79.25 ± 0.15	40	76.81 ± 0.20	41.04 ± 0.01
5	50.19 ± 0.14	60	59.59 ± 0.17	21.13 ± 0.07
6	39.94 ± 0.30	80	44.84 ± 0.13	12.20 ± 0.22

**TABLE 5:** Survival fraction of Vortioxetine and/or gemcitabine in AsPC-1.

Vortioxetine		Gemcitabine		Combination
Conc. (μM)	SF (%)	Conc. (nM)	SF (%)	SF (%)
8	75.97% ± 0.10%	200	77.99 ± 0.14	59.18 ± 0.11
9	72.45% ± 0.17%	400	66.22 ± 0.22	50.41 ± 0.16
10	66.32% ± 0.02%	600	59.81 ± 0.11	45.28 ± 0.14
11	61.73% ± 0.14%	800	56.73 ± 0.17	38.70 ± 0.31

To further validate the long-term synergistic antiproliferative effect, we performed colony formation assays after 14 days of drug treatment. In Capan-1 cells, the colony formation rates of vortioxetine and gemcitabine alone were 78.61% ± 0.13% and 78.96% ± 1.43%, respectively, which decreased to 41.04% ± 0.52% in the combination group (****P < 0.0001). Similar synergistic inhibition was seen in BxPC-3 (84.90% ± 0.39% and 83.33% ± 0.78% vs. 23.70% ± 0.59%, ****P < 0.0001) and AsPC-1 cells (79.00% ± 0.59% and 83.20% ± 0.59% vs. 43.04% ± 1.38%, ****P < 0.0001). These results demonstrate that vortioxetine synergistically enhances the inhibitory effects of gemcitabine on pancreatic cancer cell proliferation.

### 3.3 Vortioxetine combined with gemcitabine promotes apoptosis

**FIGURE 3:**
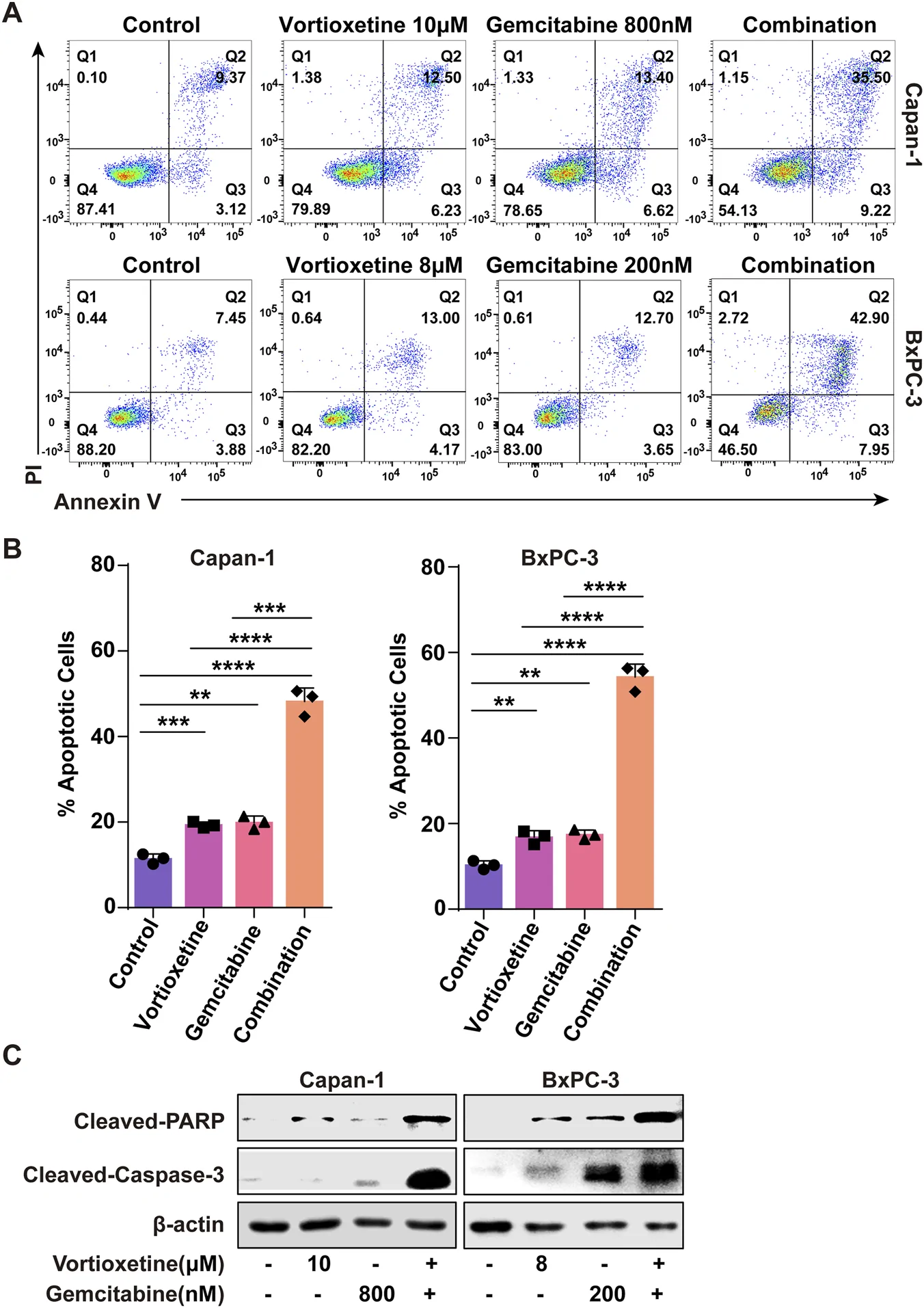
Vortioxetine enhances gemcitabine-induced apoptosis by upregulating Cleaved-PARP and Cleaved-Caspase-3 in pancreatic cancer cells. **(A)** The apoptotic rates of Capan-1 and BxPC-3 cells treated with vortioxetine alone or in combination with gemcitabine. **(B)** Statistical analysis of Capan-1 and BxPC-3 cells after 48 h of drug treatment. **(C)** Vortioxetine and gemcitabine synergistically induce apoptosis protein. The expression levels of apoptotic proteins Cleaved-PARP and Cleaved-Caspase-3 were upregulated. In **(B)** Data are presented as mean ± SD from three independent biological replicates (n = 3). Statistical significance was determined by one-way ANOVA followed by Tukey’s multiple-comparison test. **P < 0.01, ***P < 0.001, ****P < 0.0001.

To investigate the mechanism by which vortioxetine combined with gemcitabine synergistically kills tumor cells, we assessed apoptosis using Annexin V-FITC/PI double staining followed by flow cytometry. Based on the different sensitivities of the cells to the drugs and the effects of the drug combination on pancreatic cancer cell proliferation mentioned above, Capan-1 cells were treated with vortioxetine (10 μM) alone or combined with gemcitabine (800 nM), BxPC-3 cells with vortioxetine (8 μM) alone or combined with gemcitabine (200 nM), and AsPC-1 cells with vortioxetine (11 μM) alone or combined with gemcitabine (800 nM). After 48 h of treatment, apoptosis was measured. In BxPC-3 cells, the apoptosis rates were 11.33% ± 2.51% (control), 17.17% ± 10.37% (vortioxetine alone), 16.35% ± 0.38% (gemcitabine alone), and increased significantly to 50.85% ± 4.51% in the combination group (Figure 3A). Statistical analysis showed that the combination group differed significantly from the vortioxetine alone (***P < 0.001) and gemcitabine alone groups (***P < 0.001) (Figure 3B). Similarly, in Capan-1 cells, apoptosis rates were 12.49% ± 2.25% (control), 18.73% ± 3.13% (vortioxetine alone), 20.02% ± 0.40% (gemcitabine alone), and significantly increased to 44.72% ± 2.89% in the combination group (Figure 3A), with significant differences compared to single treatments (***P < 0.001 and **P < 0.01, respectively, Figure 3B).

To further confirm that the combination promotes apoptosis, apoptosis-related proteins were detected in Capan-1 and BxPC-3 cells from the different treatment groups (Figure 3C). The results showed that expression levels of cleaved-PARP and cleaved-caspase-3 were significantly higher in the combination group compared to either single treatment, consistent across all three cell lines. These findings suggest that vortioxetine combined with gemcitabine induces the expression of apoptosis-related proteins in pancreatic cancer cells, thereby promoting apoptosis and synergistically inhibiting tumor growth.

### 3.4 Vortioxetine enhances gemcitabine’s inhibition of migration and invasion

**FIGURE 4:**
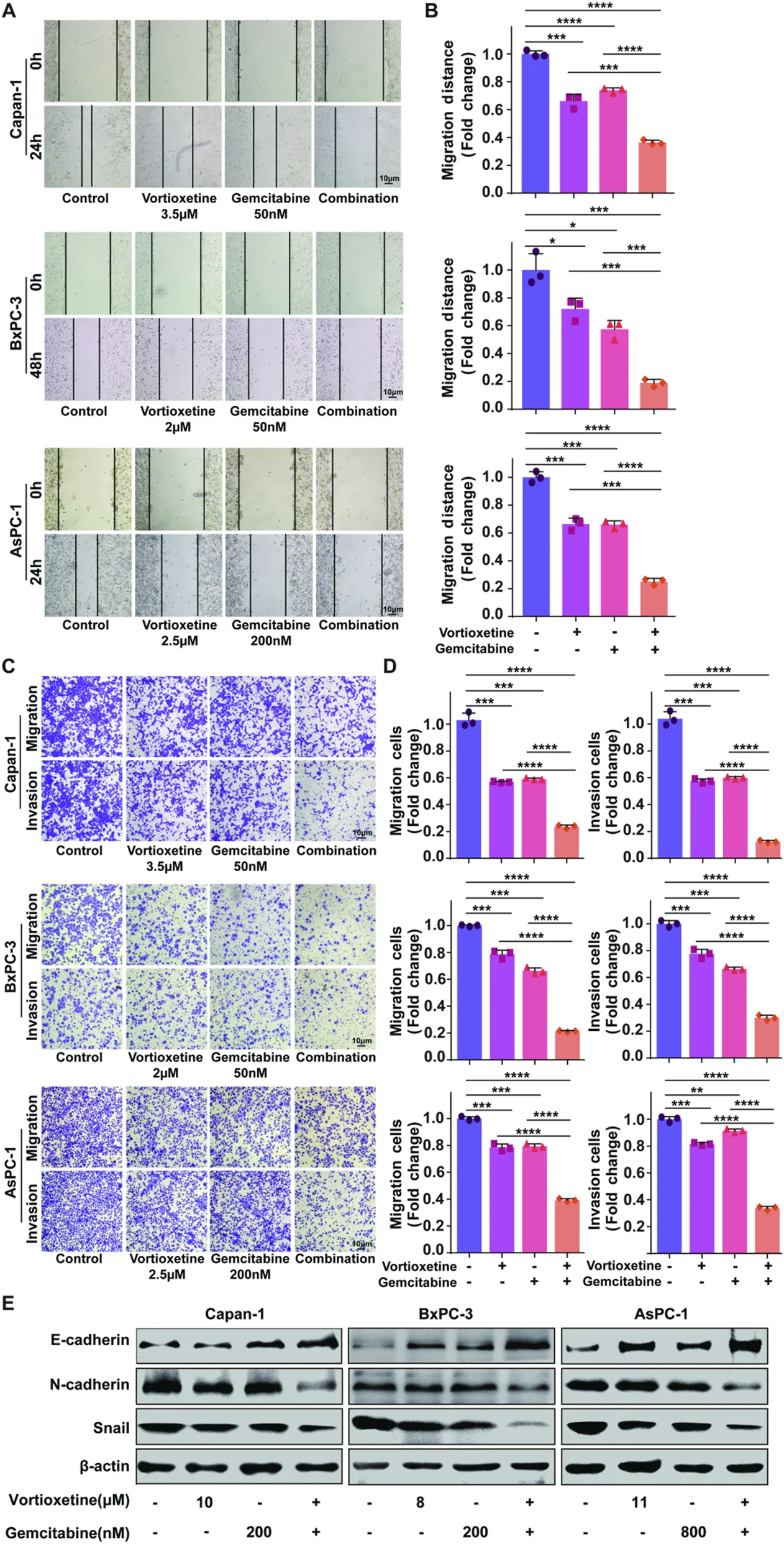
Vortioxetine enhances gemcitabine’s inhibitory effects on migration, invasion, and EMT-related protein expression in pancreatic cancer cells. **(A)** Migration ability of pancreatic cancer cells after 24 h of scratch wound assay following treatment with vortioxetine alone or in combination with gemcitabine. **(B)** Corresponding statistical analysis of pancreatic cancer cell migration in **(A)**. **(C)** Migration and invasion abilities of pancreatic cancer cells treated with vortioxetine alone or in combination with gemcitabine. **(D)** Corresponding statistical analysis of pancreatic cancer cells migration and invasion after 24 h. **(E)** Vortioxetine enhances the effect of gemcitabine on the expression of EMT-related proteins in pancreatic cancer cells. In B and **(D)**. Data are presented as mean ± SD from three independent biological replicates (n = 3). Statistical significance was determined by one-way ANOVA followed by Tukey’s multiple-comparison test. *P < 0.05, **P < 0.01, ***P < 0.001, ****P < 0.0001.

In addition to investigating the effects on tumor cell proliferation, we further examined the impact of vortioxetine combined with gemcitabine on the migration and invasion abilities of pancreatic cancer cells. Wound healing assays showed that, in Capan-1 cells, the combination of vortioxetine (3.5 μM) and gemcitabine (50 nM) significantly inhibited cell migration compared to either drug alone (****P < 0.0001, Figures 4A,B). Similarly, in BxPC-3 cells, the combination of vortioxetine (2 μM) and gemcitabine (50 nM) markedly suppressed migration (****P < 0.0001, Figures 4A,B). In AsPC-1 cells, combined treatment with vortioxetine (2.5 μM) and gemcitabine (200 nM) also significantly reduced migration (****P < 0.0001, Figures 4A,B). Consistent results were observed in transwell assays using the same drug concentrations, where combination treatment significantly suppressed both migration and invasion in all three cell lines (Figures 4C,D). These findings suggest that vortioxetine enhances gemcitabine-mediated inhibition of pancreatic cancer cell migration and invasion *in vitro*. To further investigate the underlying mechanisms, we performed western blot analysis to assess the expression of migration-related proteins (Figure 4E), using the same drug concentrations as in the apoptosis assays (Figure 3). The results showed that both vortioxetine alone and in combination with gemcitabine significantly downregulated N-cadherin and Snail expression while upregulating E-cadherin expression compared to the control group. Notably, the combination treatment resulted in the most pronounced changes, suggesting that vortioxetine combined with gemcitabine may inhibit pancreatic cancer cell migration and invasion by modulating the epithelial-mesenchymal transition (EMT) pathway.

### 3.5 Identification of MAOB as a potential target of vortioxetine

**FIGURE 5:**
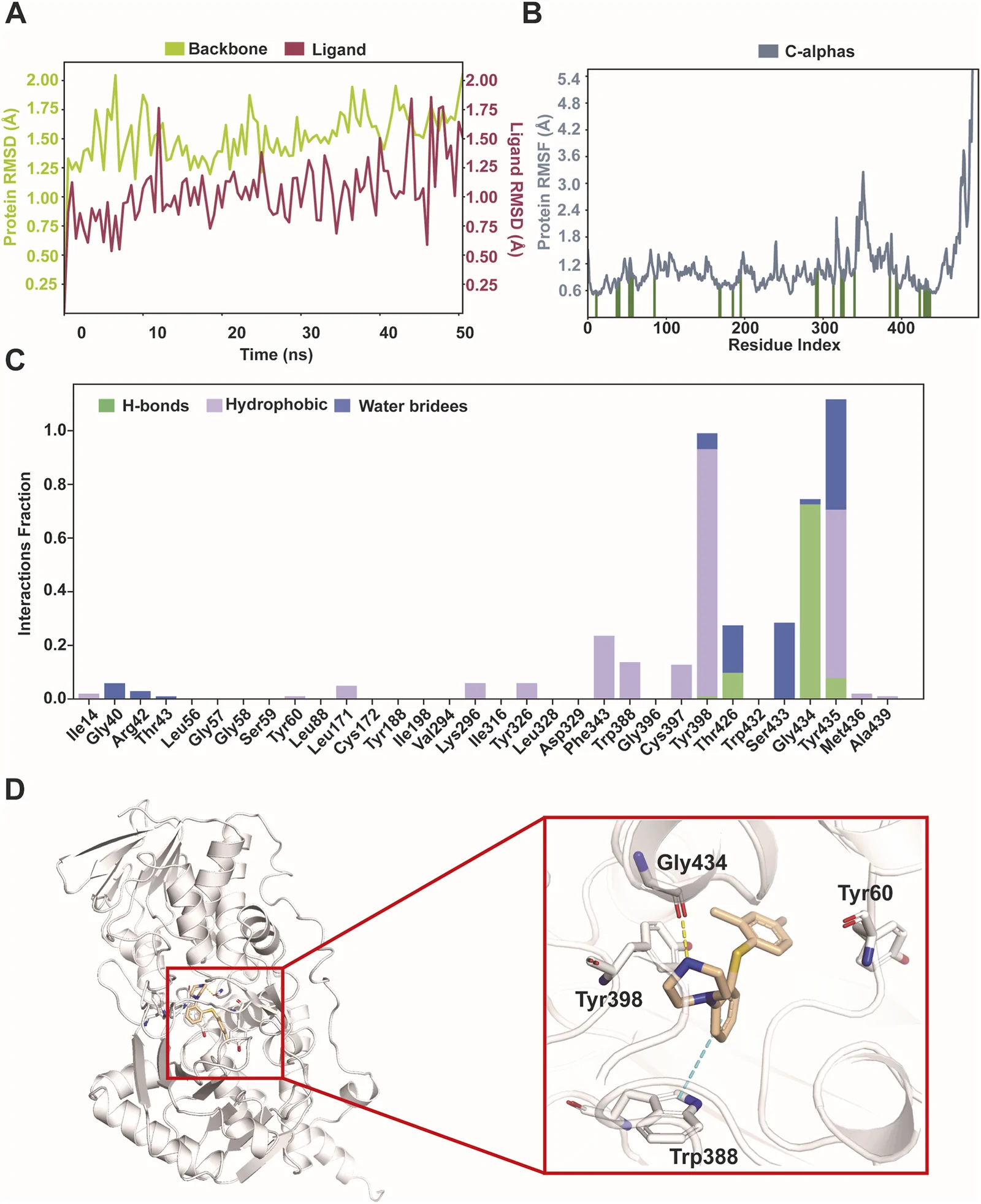
Comprehensive analysis of compound vortioxetine and MAOB interactions. **(A)** RMSD values for compound vortioxetine (red) and MAOB (green). **(B)** RMSF profile of MAOB. **(C)** Overall conformation of compound vortioxetine and MAOB binding mode. **(D)** 3D diagram of protein-ligand interactions.

To identify the key factors by which vortioxetine enhances the anti-tumor effect of gemcitabine in pancreatic cancer, we performed RNA-seq analysis comparing the Control group and the vortioxetine monotherapy group. Differentially expressed genes (DEGs) with significant changes were screened using the criterion log2 Fold Change < −2. Additionally, we imported the chemical structure of vortioxetine into the Swiss Target Prediction database to identify its potential drug targets, and used GeneCards by inputting the disease term “pancreatic cancer” to retrieve disease-related targets. By visualizing the intersection of DEGs, potential drug targets predicted by Swiss Target Prediction, and pancreatic cancer-related targets from GeneCards via Venn diagrams, we identified five overlapping genes: MAOB (P = 5.45E-06), TACR1 (P = 0.0083), HTR1B (P = 6.87E-09), SCN5A (P = 0.0002), and TERT (P = 5.47E-06) (Figures 5A,B). Further analysis revealed that MAOB was the most significantly downregulated gene among the five. Furthermore, to evaluate the clinical relevance of MAOB in pancreatic cancer, bioinformatic analysis of public GEO datasets (GSE62452 and GSE57495) demonstrated that MAOB mRNA expression was significantly upregulated in PDAC tissues compared to adjacent normal tissues (P = 0.0032, Supplementary Figure S1A). Moreover, high MAOB expression showed a trend toward worse overall survival in PDAC patients (Supplementary Figure S1B), underscoring its potential pathological significance. To validate this finding, we performed RT-qPCR to assess the mRNA expression levels of these five genes in BxPC-3 and AsPC-1 cells. The results showed that only MAOB exhibited significant downregulation in both cell lines after treatment with varying concentrations of vortioxetine (Figure 5C), consistent with the RNA-seq results. Western blotting further confirmed that vortioxetine at 8 μM and 10 μM significantly reduced MAOB protein levels (Figure 5D), consistent with the mRNA expression results. To evaluate target isoform specificity, we also examined the expression of MAOA (monoamine oxidase A) following vortioxetine treatment. The results showed no statistically significant change in MAOA mRNA levels (Supplementary Figure S2), confirming that vortioxetine preferentially downregulates the MAOB isoform in pancreatic cancer cells. Based on these findings, we hypothesize that MAOB may serve as a key target mediating the enhanced anti-tumor effect of vortioxetine combined with gemcitabine. We further examined the effect of vortioxetine on the thermal stability of MAOB protein at temperatures ranging from 40 °C to 64 °C (Figure 5E). Compared with the vortioxetine-treated group, the control group exhibited a more rapid and pronounced decrease in MAOB protein levels within this temperature range, suggesting that the thermal stability of MAOB was compromised. In contrast, in the vortioxetine-treated group, the decrease in MAOB protein levels was slower and less pronounced across the same temperature range. Meanwhile, the expression of the internal control β-actin remained largely unchanged under different temperature conditions in both groups (Figure 5E). These results indicate that vortioxetine treatment enhances the thermal stability of MAOB protein, suggesting a potential physical interaction or conformational stabilization with MAOB.

**FIGURE 6:**
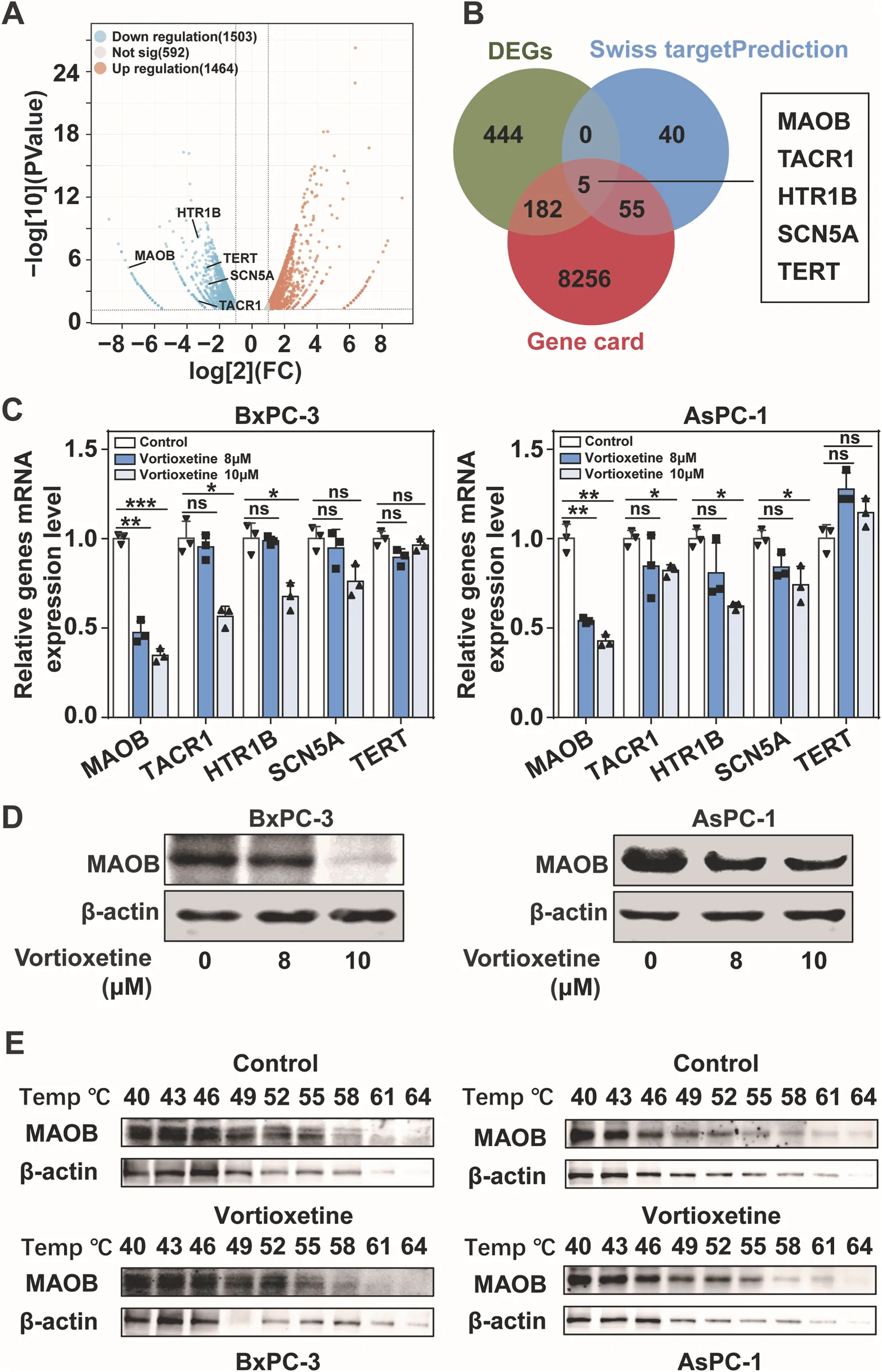
Vortioxetine downregulates MAOB expression in pancreatic cancer cells. **(A)** Volcano plot of RNA-seq results for BxPC-3 cells treated with vortioxetine alone for 24 h. **(B)** Venn diagram integrating DEGs, SwissTargetPrediction, and GeneCards identified five potential target genes: MAOB, TACR1, HTR1B, SCN5A, and TERT. Vortioxetine inhibited MAOB expression in a concentration-dependent manner. **(C,D)** RT-qPCR and western blot validation in BxPC-3 and AsPC-1 cells treated with vortioxetine confirmed reduced MAOB mRNA and protein levels. **(E)** MAOB protein stability in BxPC-3 and AsPC-1 cells was further assessed under different temperature gradients. In **(A,C)** Data are presented as mean ± SD from three independent biological replicates (n = 3). Statistical significance was determined by one-way ANOVA followed by Tukey’s multiple-comparison test. *P < 0.05, **P < 0.01, ***P < 0.001, ****P < 0.0001.

To enhance our understanding of how the compound vortioxetine interacts with the MAOB protein (PDB ID: 1S3E), we conducted computational docking and molecular dynamics (MD) simulations. We initially simulated the MAOB-vortioxetine complex for 50 ns and calculated the root mean square deviation (RMSD) for both the protein backbone and vortioxetine throughout the trajectory. As illustrated in Figure 6A, the system reached a dynamic equilibrium, reflected in the stable RMSD values. Furthermore, root mean square fluctuation (RMSF) analysis pinpointed residues like Phe343 and Trp388, which displayed increased mobility, indicating flexible regions within the protein (Figure 6B). A closer look at the key amino acids revealed that Tyr398, Gly434, and Tyr435 were essential for the interaction between MAOB and vortioxetine (Figure 6C). Additionally, our analysis showed that the piperazine group of vortioxetine formed a hydrogen bond with Gly434, while the benzene ring engaged in π-π stacking interactions with Trp388 (Figure 6D). In conclusion, our detailed computational investigation revealed a likely binding mechanism in which vortioxetine interacts with the MAOB protein through significant hydrogen bonds and π-π stacking interactions, contributing to the overall stability of the MAOB-vortioxetine complex.

### 3.6 Downregulation of MAOB enhances the sensitivity to gemcitabine

**FIGURE 7:**
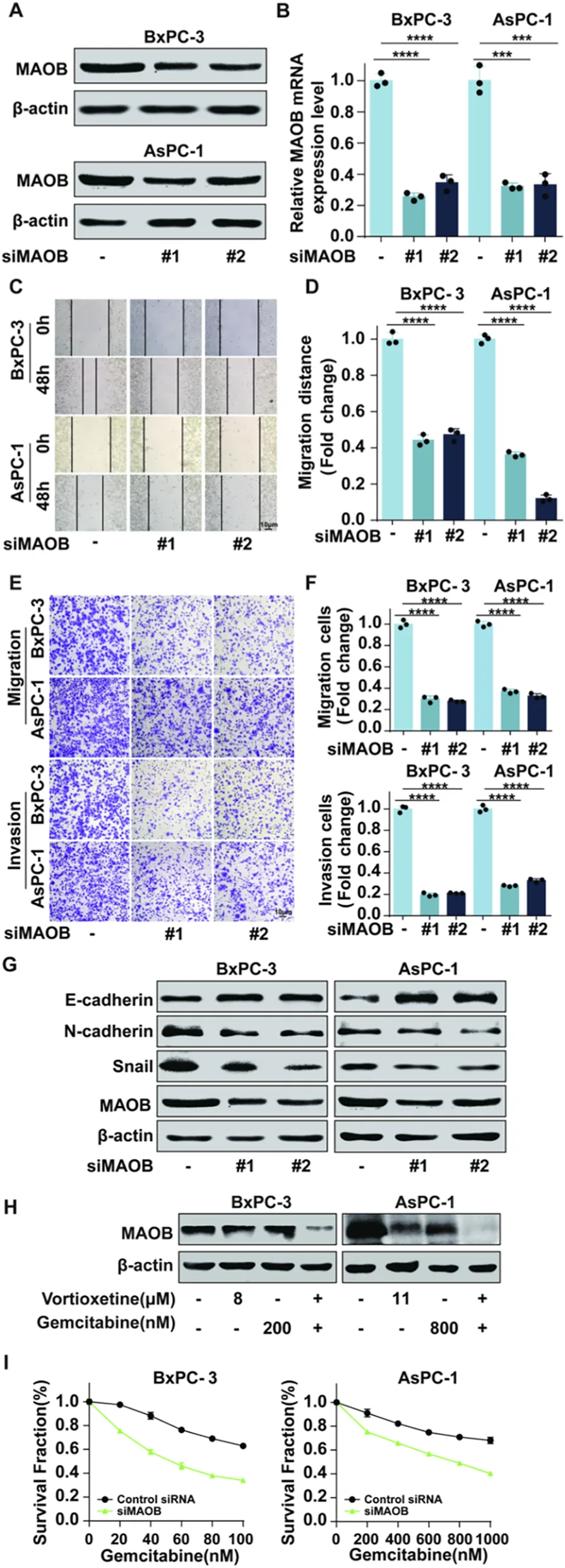
MAOB knockdown inhibits pancreatic cancer cell migration, invasion, EMT, and enhances sensitivity to gemcitabine. **(A,B)** Silencing the MAOB gene significantly reduced both protein and mRNA expression levels of MAOB in pancreatic cancer cells BxPC-3 and AsPC-1. **(C–F)** Knockdown of MAOB markedly inhibited the wound-healing ability of these cells, as well as their migration and invasion capabilities. **(G)** Suppression of MAOB expression led to downregulation of EMT-related proteins N-cadherin and Snail, accompanied by upregulation of E-cadherin. **(H)** Treatment with vortioxetine combined with gemcitabine significantly decreased MAOB protein expression in both BxPC-3 and AsPC-1 cells. **(I)** MAOB silencing enhanced the sensitivity of pancreatic cancer cells to varying concentrations of gemcitabine, indicating a potential role in overcoming chemoresistance. In B, D, and **(F)** Data are presented as mean ± SD from three independent biological replicates (n = 3). Statistical significance was determined by one-way ANOVA followed by Tukey’s multiple-comparison test. ***P < 0.001, ****P < 0.0001.

To further investigate the role of MAOB in the progression of pancreatic cancer, we used siRNA to knockdown MAOB expression in two pancreatic cancer cell lines. The results showed that both siMAOB-#1 and siMAOB-#2 significantly reduced MAOB mRNA and protein levels compared to the NC group (Figures 7A,B). After successfully silencing MAOB, we assessed its effect on pancreatic cancer cell migration and invasion. Wound healing assays revealed that MAOB knockdown inhibited the wound closure ability of BxPC-3 and AsPC-1 cells compared to the NC group (Figures 7C,D). Furthermore, transwell assays confirmed that MAOB silencing significantly suppressed the migration and invasion abilities of pancreatic cancer cells (Figures 7E,F). Western blot analysis showed that silencing MAOB reduced the expression of N-cadherin and Snail while increasing E-cadherin levels (Figure 7G), suggesting that MAOB knockdown may inhibit the metastatic potential of pancreatic cancer cells via the EMT pathway.

Collectively, these results indicate that vortioxetine downregulates MAOB at both mRNA and protein levels, and that MAOB downregulation reduces the migration and invasion capabilities of pancreatic cancer cells *in vitro*. Additionally, the combination of vortioxetine and gemcitabine further decreased the migration and invasion of pancreatic cancer cells, implying that vortioxetine may enhance gemcitabine’s antitumor effects by targeting MAOB. To verify the hypothesis, we compared MAOB protein levels between the monotherapy and combination groups and found that MAOB expression was significantly lower in the combination group (Figure 7H). Moreover, after MAOB knockdown, treatment with different concentrations of gemcitabine (0, 20, 40, 60, 80, 100 nM for BxPC-3 cells; 0, 200, 400, 600, 800, 1,000 nM for AsPC-1 cells) revealed that MAOB silencing significantly increased the sensitivity of pancreatic cancer cells to gemcitabine compared to the control siRNA group (Figure 7I). These findings suggest that vortioxetine may enhance the antitumor effect of gemcitabine against pancreatic cancer by inhibiting MAOB.

### 3.7 Vortioxetine enhances gemcitabine’s inhibition of xenograft tumor growth

Finally, to evaluate the antitumor effect of vortioxetine combined with gemcitabine *in vivo*, we selected BxPC-3 cells, which demonstrated a significant combination effect and high tumorigenic efficiency, as the xenograft tumor model. An appropriate number of cells were subcutaneously injected into six-week-old BALB/c nude mice. When the tumor volume reached approximately 80 mm^3^, the mice were randomly divided into four groups: negative control, vortioxetine monotherapy, gemcitabine monotherapy, and vortioxetine combined with gemcitabine. Tumor length, width, and body weight were measured every other day, and relative tumor volume (RTV) was calculated.

**FIGURE 8:**
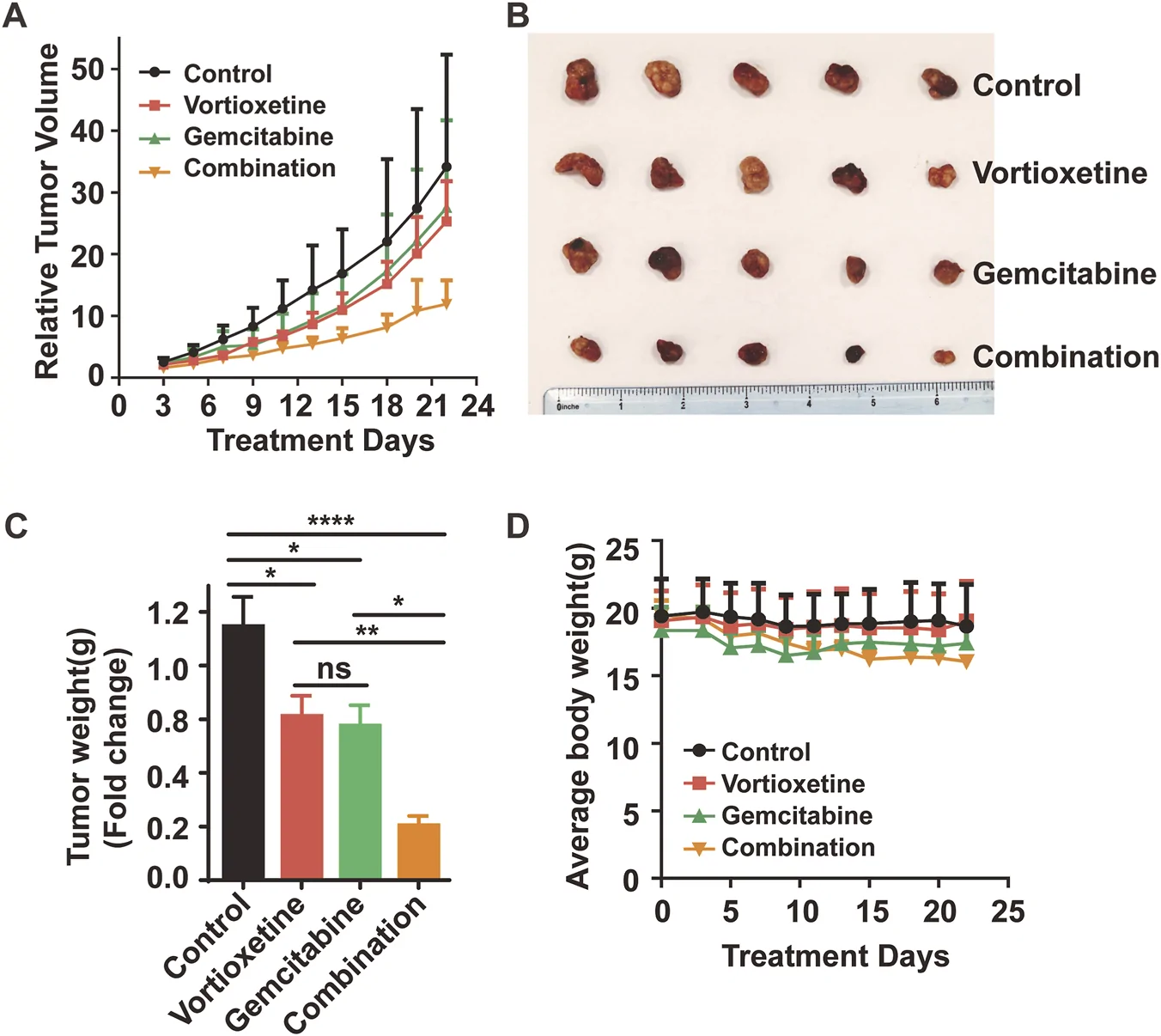
Vortioxetine and gemcitabine inhibit the growth of pancreatic cancer cells *in vivo*. **(A)** RTV curve of nude mice. **(B)** Body weight monitoring of mice throughout the treatment period. **(C)** Representative images of subcutaneous tumors in nude mice. **(D)** Tumor weight measurements. In A, B, C, and **(D)** Data are presented as mean ± SD; n = 5 mice per group. Statistical significance was determined by one-way or two-way ANOVA followed by multiple-comparison tests, as appropriate. ns. not significant, *P < 0.05, **P < 0.01, ****P < 0.0001.

The results showed that, compared to the control group, the monotherapy groups did not exhibit a significant reduction in RTV, whereas the combination group significantly inhibited tumor growth (Figure 8A). Additionally, there was no significant difference in body weight among the groups (Figure 8B), suggesting preliminary safety of the combination treatment. Further analysis of excised xenograft tumors revealed that both the tumor images and tumor weights confirmed that vortioxetine combined with gemcitabine markedly suppressed tumor growth in nude mice (Figures 8C,D).

Together with the *in vitro* results showing proliferation inhibition, these findings indicate that vortioxetine combined with gemcitabine exerts synergistic antitumor effects on pancreatic cancer cells both *in vitro* and *in vivo*.

## 4 Discussion

Gemcitabine is a broad-spectrum chemotherapeutic agent widely used in the treatment of various malignancies, including pancreatic cancer (Wainberg et al., 2023), non-small cell lung cancer (Zhong et al., 2023), breast cancer (Singh et al., 2022), cholangiocarcinoma (Kelley et al., 2023), and bladder cancer (Pfister et al., 2022). According to the latest NCCN Guidelines for Pancreatic Cancer (2024 edition), chemotherapy remains the standard clinical treatment and the main therapeutic approach for advanced pancreatic cancer. Given the highly chemoresistant nature of pancreatic cancer, combination therapy is considered an effective strategy to overcome treatment resistance. The guidelines recommend several gemcitabine-based first-line chemotherapy regimens, including: 1) FOLFIRINOX or modified FOLFIRINOX, 2) gemcitabine combined with nab-paclitaxel, and 3) liposomal irinotecan combined with 5-FU, leucovorin, and oxaliplatin (NALIRIFOX). Other recommended options include gemcitabine monotherapy, gemcitabine plus capecitabine, gemcitabine plus erlotinib, and GTX regimens. However, due to individual differences among pancreatic cancer patients, long-term high-dose chemotherapy with gemcitabine often results in severe toxicity and adverse effects, leading to treatment discontinuation in many patients. Therefore, there is an urgent need to develop more effective therapeutic strategies with reduced toxicity.

Serotonin (5-HT) has been implicated in the development and progression of pancreatic cancer. For instance, Gurbuz et al. (Gurbuz et al., 2014), found that downregulation of 5-HT1B and 5-HT1D receptors suppresses proliferation and invasion of pancreatic cancer cells. Other studies have reported that prolonged exposure to 5-HT enhances EZH2 expression, which in turn upregulates TPH1 and 5-HT7 expression, contributing to chemoresistance in pancreatic cancer. Targeting the EZH2-TPH1-5-HT7 axis reverses drug resistance (Chaudhary et al., 2021), highlighting the therapeutic potential of targeting 5-HT receptors. Vortioxetine, a multimodal serotonergic antidepressant with serotonin transporter inhibitory activity and multiple 5-HT receptor-modulating properties, has demonstrated good tolerability and safety in clinical practice. Recent studies have indicated its anti-tumor potential. For example, vortioxetine hydrobromide has been shown to inhibit gastric cancer cell proliferation via the JAK2/SRC-STAT3 pathway (Li et al., 2023), and to induce apoptosis and autophagy through the PI3K-AKT pathway (Lv et al., 2020), thereby reducing tumor cell proliferation, migration, and invasion.

To date, the effect of vortioxetine alone or in combination with gemcitabine in pancreatic cancer has not been reported. Our study demonstrated that vortioxetine inhibits pancreatic cancer cell proliferation in a concentration- and time-dependent manner, with increasing drug concentrations and exposure times resulting in more pronounced anti-proliferative effects. Additionally, vortioxetine inhibited the migration, invasion, and wound-healing capacity of pancreatic cancer cells. However, the IC50 values of vortioxetine in different pancreatic cancer cell lines ranged from 3 to 11 μM, indicating cytotoxicity only at relatively high concentrations, with significant inhibition of migration and invasion observed mainly at high doses. This suggests that vortioxetine alone has limited anti-tumor potential.

Given that gemcitabine is a first-line chemotherapeutic agent for advanced pancreatic cancer and that resistance limits its clinical efficacy, we further explored whether vortioxetine could sensitize pancreatic cancer cells to gemcitabine. Our results showed that the combination of vortioxetine and gemcitabine exhibited synergistic anti-proliferative effects in pancreatic cancer cells. Both scratch and transwell assays confirmed that combination treatment significantly inhibited cell migration and invasion. *In vivo* xenograft models also demonstrated superior tumor growth inhibition with the combination therapy. Collectively, our data indicate that vortioxetine enhances gemcitabine’s anti-tumor activity both *in vitro* and *in vivo*, suggesting a promising combinational strategy for pancreatic cancer treatment.

Chemotherapy commonly exerts anti-tumor effects by inhibiting proliferation and promoting apoptosis (Cohen, 1997). Caspase-3 is a key executioner in apoptosis, and cleaved caspase-3 is a hallmark of its activation (Kesavardhana et al., 2020). Cleavage of PARP is also an important marker of apoptosis (Oliver et al., 1998). In our study, combination therapy promoted the activation of cleaved caspase-3 and cleavage of the DNA repair-related protein PARP, leading to apoptotic cell death. Moreover, EMT, a process closely associated with tumor metastasis and invasion (Pastushenko and Blanpain, 2019), involves transcription factors such as snail (Lan et al., 2022), which suppresses E-cadherin expression and promotes EMT. Our findings indicated that combination treatment suppressed Snail, downregulated N-cadherin, and upregulated E-cadherin, suggesting inhibition of EMT as a mechanism of reduced invasiveness.

To further explore the underlying mechanism of vortioxetine-enhanced gemcitabine sensitivity, we conducted RNA-seq and identified MAOB as a potential target, with significantly downregulated mRNA levels following vortioxetine treatment. These results were validated by RT-qPCR and Western blot, showing reduced MAOB expression at both mRNA and protein levels, indicating that vortioxetine may target MAOB to potentiate gemcitabine’s anti-pancreatic cancer activity. MAOB, a member of the flavin monoamine oxidase family, primarily catalyzes oxidative deamination of amines, including exogenous amines and neurotransmitters (Sturza et al., 2013). It differs from monoamine oxidase A in terms of substrate affinity, tissue distribution, and inhibitor specificity. Although MAOB is mainly targeted in Parkinson’s disease (Gray et al., 2022; Wu et al., 2021), recent studies have revealed differential MAOB expression in various cancers and normal tissues (Xu et al., 2019; Zhang et al., 2021), with neurotransmitters as its substrates implicated in cancer pathogenesis (Zahalka et al., 2020). MAOB has been identified as a key gene affecting proliferation and metastasis in lung cancer (Shi et al., 2024) and has also been associated with prostate cancer progression. In our study, vortioxetine inhibited MAOB expression in a concentration-dependent manner. Molecular docking analyses demonstrated that vortioxetine can stably bind to MAOB, with key interactions involving hydrogen bonding with Gly434 and π-π stacking with Trp388. These findings suggest that vortioxetine may directly interact with MAOB to regulate its function, providing mechanistic insight into its potential antitumor activity. Silencing MAOB significantly inhibited pancreatic cancer cell migration and invasion. Furthermore, combination treatment with vortioxetine and gemcitabine maximally reduced MAOB expression. Importantly, knockdown of MAOB enhanced gemcitabine’s anti-tumor effects, supporting the hypothesis that vortioxetine sensitizes pancreatic cancer cells to gemcitabine by targeting MAOB, but the precise signaling pathways involved remain unclear. Additionally, the molecular mechanism by which vortioxetine interacts with MAOB has not yet been investigated. Further studies are required to elucidate how vortioxetine modulates MAOB and to clarify the pathways involved in enhancing gemcitabine efficacy.

Although MAOB knockdown phenocopies vortioxetine treatment, a limitation of the present study is the lack of ectopic MAOB rescue experiments and direct recombinant enzyme catalytic assays. Future studies utilizing plasmid-mediated MAOB overexpression and enzymatic cleavage kinetics will be required to confirm whether MAOB is functionally indispensable for vortioxetine-mediated chemosensitization.

Furthermore, the *in vitro* IC_50_ values of vortioxetine in PDAC cells (3–11 μM) exceed typical plasma concentrations achieved in clinical psychiatric treatment. Therefore, vortioxetine should be considered a lead scaffold for optimization or targeted delivery formulations rather than an immediate standalone repositioned agent.

Given vortioxetine’s multimodal activity across 5-HT receptor subtypes, future work using specific 5-HT receptor antagonists/agonists will help clarify the exact cross-talk between cell-surface serotonergic receptors and mitochondrial MAOB downregulation.

## 5 Conclusion

In conclusion, our findings suggest that vortioxetine suppresses proliferation, migration, invasion, and promotes apoptosis in pancreatic cancer cells, and enhances gemcitabine sensitivity via targeting MAOB. The combination of vortioxetine and gemcitabine may represent a novel therapeutic strategy for pancreatic cancer, providing a rationale for further clinical exploration of vortioxetine’s anti-tumor potential.

## Data Availability

The original contributions presented in the study are publicly available. This data can be found here: https://github.com/ZXL700/MAOB.git.

## Supplementary material

Supplementary Figure S1 Clinical relevance of MAOB in pancreatic ductal adenocarcinoma 667 (PDAC).

Supplementary Figure S2 Effect of vortioxetine on MAOA expression in pancreatic cancer cells.
